# Mutualism between Gut-Borne Yeasts and Their Host, *Thaumatotibia* *leucotreta*, and Potential Usefulness in Pest Management

**DOI:** 10.3390/insects13030243

**Published:** 2022-02-28

**Authors:** Marcel van der Merwe, Michael D. Jukes, Caroline Knox, Sean D. Moore, Martin P. Hill

**Affiliations:** 1 Department of Biochemistry and Microbiology, Rhodes University, P.O. Box 94, Makhanda 6140, South Africa; m.jukes@ru.ac.za (M.D.J.); caroline.knox@ru.ac.za (C.K.); 2 Centre for Biological Control, Department of Zoology and Entomology, Rhodes University, P.O. Box 94, Makhanda 6140, South Africa; seanmoore@cri.co.za (S.D.M.); m.hill@ru.ac.za (M.P.H.); 3 Citrus Research International, P.O. Box 5095, Walmer, Gqeberha 6013, South Africa

**Keywords:** insect–microbe interaction, mutualism, false codling moth, yeast

## Abstract

**Simple Summary:**

The false codling moth is an indigenous pest of the citrus industry in southern Africa. It is a major threat as it can cause substantial fruit damage through larval feeding. The microorganisms associated with insects are often studied for their potential to kill their host instead of how they could aid them. However, in recent years, this aspect of their interaction has received much attention. The codling moth has been shown to have a close relationship with the naturally occurring yeast found within their digestive tract. These beneficial yeasts help the adult female moths locate a suitable host for egg laying. During their larval phase, these yeasts help them develop faster and increase their chance of survival. These interactions could be manipulated for the purposes of biological control, as one might be able to combine these yeasts with existing biological control agents to improve their effectiveness. These yeasts may also provide additional methods for monitoring field populations of insect pests. In this study, we identified yeasts that occur naturally in the guts of false codling moth larvae and investigated whether any of them are beneficial to their host.

**Abstract:**

*Thaumatotibia* *leucotreta* is endemic to southern Africa and is highly significant for various fruit industries, including the South African citrus industry, due to its classification as a phytosanitary pest. Mutualistic associations between *C*. *pomonella*, closely related to *T*. *leucotreta*, and yeasts have previously been described and reported to reduce larval mortality and enhance larval development. Here, we determined which yeast species occur naturally in the gut of *T*. *leucotreta* larvae and investigated whether any of the isolated yeast species affect their behaviour and development. Navel oranges infested with *T*. *leucotreta* larvae were collected from geographically distinct provinces in South Africa, and the larvae were processed for analysis of naturally occurring associated yeasts. Six yeast species were isolated and identified from the guts of these *T*. *leucotreta* larvae via PCR amplification and sequencing of the ITS region of rDNA and D1/D2 domain of large ribosomal subunit. Larval development and attraction assays were conducted, and *T*. *leucotreta* larvae that fed on Navel oranges inoculated with yeast had accelerated developmental periods and reduced mortality rates. Neonate *T. leucotreta* were also attracted to YPD broth cultures inoculated with yeast for feeding. Oviposition preference assays were conducted with adult *T*. *leucotreta* females. Navel oranges inoculated with yeast were shown to influence the oviposition preference of adult females. Yeasts harbour the potential for use in biocontrol, especially when combined with other well-established control methods. This study provides a platform for future research into incorporating yeast with current biological control agents as a novel option for controlling *T*. *leucotreta* in the field.

## 1. Introduction

Microbes are the most abundant living organisms on earth, and apart from being found within the general environment, they are also known to colonise other living organisms such as insects [1]. The relationships between insects and microorganisms are often not overtly pathogenic but rather beneficial or even required [2]. They vary considerably and range from accidental encounters to functional ones, such as locating an attractive food source or providing essential nutrients missing from their diet [3,4]. Interactions between insects and microbes with a mutualistic relationship are greater than those that interact by chance, as they have evolved together [5,6]. Microbes are predominantly localised to the external cuticle, gut, hemocoel and cells of insects, as these regions offer the most favourable conditions for establishment [1]. Microbes ingested and hosted within the insect gut can strongly impact insect survival [7,8]. The makeup of these microbial communities mainly comprise bacteria, archaea and eukaryotic microorganisms [9,10]. The microbiota harboured within insects is generally distinct from the microorganisms in the external environment [2]. Microbial populations are usually concentrated in the digestive tract of most insects, particularly the hindgut, as it is a benign environment that provides access to nutrients and protection from the various stresses associated with the external environment [3]. The role of microbial communities in insect–plant interactions is essential and can favourably impact their ecology, evolution and behaviour [11,12,13,14,15]. Several insect orders have been reported to harbour persistent microbial communities [16]. Associations between Lepidoptera and microbes have rarely been described even though they are the second-most diverse insect order with some of the most devastating agricultural pests worldwide [17,18].

Unlike their bacterial counterparts, interactions between insects and yeasts have been understudied, despite their importance to insect fitness and behaviour [9,19,20]. The search for associations between Lepidoptera and yeasts was ignited by studies conducted on *Drosophila melanogaster* (Meigen) (Diptera: Drosophilidae) [21]. The presence, type and amount of yeast within their diet affect their behaviour, development and fitness [14,22,23]. Yeasts were also shown to be primarily responsible for influencing host locating and ovipositing, not plant cues as once thought [24]. Yeasts have proven to be an essential nutritional source for insect larval development and to influence neonate larval feeding and behaviour [25]. For *Cydia pomonella* (Linnaeus) (Lepidoptera: Tortricidae), commonly known as the codling moth, larvae are closely associated with yeast from the genus *Metschnikowia* [26]. These yeasts were reported to be crucial in enhancing the development of *C*. *pomonella* larvae and reducing mortality. Additionally, the metabolites produced by yeasts contributed towards adult *C*. *pomonella* moths recognising and finding their host plant [26]. Identifying this mutualistic association between *C*. *pomonella* larvae and yeasts from the genus *Metschnikowia* has led to the development of novel biocontrol strategies [27,28,29].

*Thaumatotibia leucotreta* (Meyrick) (Lepidoptera: Tortricidae), commonly known as the false codling moth, is a phytophagous insect, endemic to southern Africa [30]. It is important to various fruit industries, including the South African citrus industry, due to its classification as a phytosanitary pest by several international markets [31]. The pest is capable of causing significant financial loss through larval feeding. However, in recent years, it has been effectively suppressed, through much improved management [32,33,34]. There are several biological control agents available for use against *T*. *leucotreta* in South Africa [33,35]. Most notably, however, is the betabaculovirus, Cryptophlebia leucotreta granulovirus (CrleGV), which is an effective microbial control agent against *T*. *leucotreta* [36]. It has previously been demonstrated, in laboratory assays and field trials, that combining yeast with baculoviruses has resulted in the significant increase in larval mortality [27,28]. This indicates that yeasts harbour the potential for use in biocontrol, especially when combined with other well-established control methods. This has, however, only been reported in *C*. *pomonella* and not for *T*. *leucotreta*.

Herein, we aimed to determine which yeast species occur naturally in the gut of *T*. *leucotreta* larvae from geographically distinct citrus-producing regions across South Africa and to examine whether any isolated yeast species affected the behaviour and development of neonate larvae, as well as the oviposition preference of adult female moths. These yeasts could potentially represent rich resources of new biological agents for the control of *T*. *leucotreta* when combined with CrleGV.

## 2. Materials and Methods

### 2.1. Yeast Isolation and Purification

**Figure 1:**
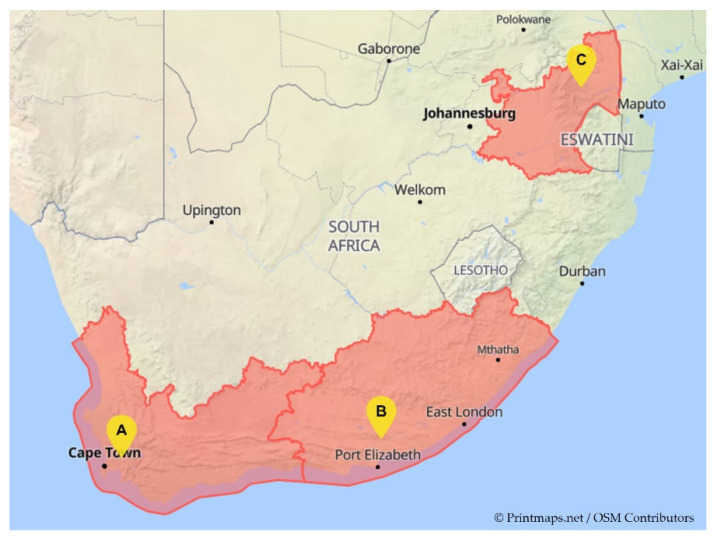
Citrus-producing regions across South Africa where *T*. *leucotreta* larvae were collected: (**A**) Stellenbosch in Western Cape, (**B**) Addo in Eastern Cape and (**C**) Nelspruit in Mpumalanga.

Ten Navel oranges (*Citrus sinensis* L. Osbeck) infested with *T*. *leucotreta* larvae were collected from one orchard in each of three geographically distinct citrus-producing regions in South Africa, namely Stellenbosch (Western Cape), Addo (Eastern Cape) and Nelspruit (Mpumalanga), between March and June of 2018 (Figure 1).

The extracted larvae (*n* = 10) were surface-sterilised by rinsing in 10% NaOCl, followed by a rinse in 70% ethanol solution (*v*/*v*) and finally double-distilled H_2_O (ddH_2_O). Yeasts were isolated from the digestive tract of *T*. *leucotreta* larvae according to Witzgall et al. [26]. Larvae were stored at 4 °C for 2 hours (h) to immobilise them. They were then dissected with a sterile razor blade in a fume hood. A sterilised inoculating loop was used to collect the gut content, which was then homogenised with a glass rod in an Eppendorf tube. The homogenate was then streaked onto a Yeast extract Peptone Dextrose (YPD) agar (Sigma-Aldrich, St. Louis, MO, USA) plate containing 40 units/mL of penicillin (Pen) and 40 µg/mL of streptomycin (Strep) (Thermo Fisher Scientific, Waltham, MA, USA). Additionally, the homogenate was diluted in ddH_2_O, filtered through muslin cloth and spread onto a Pen/Strep YPD agar plate. Plates were incubated at 27 °C for 48 h under aerobic conditions, with emerging yeast colonies being selected and discontinuously streaked on Pen/Strep YPD agar plates. This process was conducted twice to obtain pure colonies.

### 2.2. Yeast Identification

Yeast isolates were grown overnight in YPD medium containing 40 units/mL of Pen and 40 µg/mL of Strep at 27 °C while shaking (180 rpm). Genomic DNA (gDNA) was extracted using the YeaStar™ Genomic DNA Kit (Zymo Research, Irvine, CA, USA) per the manufacturer’s instructions. The concentration of gDNA was determined using a NanoDrop 2000 spectrophotometer (Thermo Fisher Scientific, Waltham, MA, USA). The amplicons of the intended targeting region were amplified using 50 ng of gDNA template with the site-specific primers (Table S1) for the internal transcribed spacer (ITS) region and D1/D2 domain of the LSU of ribosomal DNA (rDNA) [37]. PCR amplification was carried out using a SimpliAmp™ Thermal Cycler (Thermo Fisher Scientific, Waltham, MA, USA) under the following cycle parameters: 94 °C for 5 min followed by 30 cycles of 95 °C for 30 s, 55 °C for 45 s and 72 °C for 45 s. A final elongation cycle of 72 °C for 10 min was used [26]. Amplicons were sequenced by Inqaba Biotechnical Industries (Pty) Ltd. (Johannesburg South Africa). Sequences were assembled and analysed in MEGA X [38], with any ambiguous nucleotides being corrected before the sequences were identified by comparison to the GenBank database of nonredundant sequences using BLAST.

### 2.3. Thaumatotibia leucotreta Culture

Eggs and pupae were obtained from the heterogeneous *T*. *leucotreta* culture, known as “Mixed Colony”, held at Rhodes University’s Department of Zoology and Entomology, South Africa. The colony was established in 1996 using *T*. *leucotreta* collected from Citrusdal (Western Cape Province), Zebediela (Limpopo Province) and the Eastern Cape [39]. The larvae were reared and maintained on an artificial diet consisting of maize meal, wheat germ, casein, Brewer’s yeast, nipagin, sorbic acid and distilled water [40].

### 2.4. Larval Development Assays

A modified version of the larval development assay described by Witzgall et al. [26] was used to determine the effect that yeasts had on the development of *T*. *leucotreta* larvae. Navel oranges were collected from orchards in the Sundays River Valley in the Eastern Cape Province of South Africa and stored in a 4 °C cold room to preserve the fruit until use. Oranges were sterilised in a 0.5% bleach solution, rinsed twice in ddH_2_O and allowed to air dry in a controlled-environment (CE) room at 25 °C overnight. Yeast cultures were grown in YPD medium containing 40 units/mL of Pen and 40 µg/mL of Strep for 20 h at 27 °C while shaking; cell counts were adjusted to 2 × 10^6^ cells mL^−1^ with ddH_2_O. Sterilised Navel oranges were dipped three times into a specific yeast culture, placed on a platform and allowed to air-dry in a 25 °C CE room. Five newly hatched *T*. *leucotreta* neonates were then placed onto each Navel orange and maintained in a 25 °C CE room for 35 days (d). Cotton wool was placed between the Navel oranges 10 d later to provide emerging 5th instars with a pupation site. Thereafter, the cotton wool was checked daily, and the number of pupated *T*. *leucotreta* larvae was recorded. After 35 d, each Navel orange was dissected to check for any remaining *T*. *leucotreta* larvae. Assays were conducted on four dates, with nine Navel oranges per treatment.

### 2.5. Larval Feeding Assay

Two-choice bioassays were used to assess neonate *T*. *leucotreta* yeast feeding and behaviour, adapted from Ljunggren et al. [41]. Yeast cultures were grown as previously described, with cell counts adjusted to 1.5 × 10^7^ cells mL^−1^ with ddH_2_O. Two-choice bioassays were conducted whereby two 50 µL drops of yeast culture and YPD medium were pipetted across from one another, approximately 1 cm from the edge of a plastic petri dish. Red and blue colourants (Pioneer Foods, Paarl, South Africa) were used at 1:25 dilution to colour the yeast and YPD medium to distinguish between neonate *T*. *leucotreta* that fed on yeast culture, blank medium, or both. Preliminary tests did not show a bias in neonate *T*. *leucotreta* attraction to either the red or blue colourants (Figure S1) (*p* > 0.05; t = 0.2133; df = 27 (Student’s *t*-test)). Neonate *T*. *leucotreta* were starved for 24 to 48 h after hatching before being placed in the centre of a petri dish using a fine paintbrush. A glass lid was used to cover the petri dish to prevent the neonates from escaping and was left for 2 h to allow the larvae to feed. The colouration of the neonate’s gut was then observed under a dissecting microscope. Twenty-two independent replicates with 10 *T*. *leucotreta* neonates were performed for each yeast.

### 2.6. Oviposition Preference Assays

A modified version of the ovipositional preference trials described by Love et al. [42] was used to determine the oviposition preference of adult *T*. *leucotreta* females. *Thaumatotibia leucotreta* female and male pupae were kept in separate vials. Once moths had eclosed, they were paired together, within 24 h, and allowed to copulate for 48 h in a 25 mL glass vial, plugged with cotton wool moistened in a 10% sugar water solution. Pairs were kept in a 25 °C CE room with 30–60% relative humidity (RH) under light conditions. Trials were conducted in a 25 °C CE room with 30–60% RH under a day/night light cycle of 12 h of light and 12 h of dark. Navel oranges were collected and stored as previously described and removed from cold storage 1 d prior to being used in the ovipositional preference trial. The fruits were checked for *T*. *leucotreta* eggs, those with eggs being discarded, before being thoroughly washed in a 0.5% NaOCl solution, rinsed twice in ddH_2_O and allowed to air dry in a CE room at 25 °C overnight. Yeast cultures were grown as previously described, with counts adjusted to 2 × 10^6^ cells mL^−1^ with ddH_2_O. Sterilised Navel oranges were dipped three times into a specific treatment and allowed to air-dry in a 25 °C CE room. Treated Navel oranges were then placed onto 4 cm tall plastic platforms 15 cm apart in a plastic container (60 × 40 × 40 cm) covered with muslin cloth. A single pair of mated adults was then released into the container. The eggs oviposited on the Navel oranges were counted after 48 h. Ten ovipositional preference trials were conducted per yeast species.

### 2.7. Statistical Analysis

All statistical analyses were performed using GraphPad Prism version 9.0.0 (GraphPad Software, San Diego, CA, USA). *Thaumatotibia leucotreta* larval development assays were analysed using a Fisher’s exact test [26]. The larval feeding of *T*. *leucotreta* on yeasts was compared using a Student’s *t*-test, with the level of significance set to *p* = 0.05 [41]. The ovipositional preference trials were analysed using a Student’s *t*-test, with a significance level of *p* = 0.05 [42].

## 3. Results

### 3.1. Yeast Isolation and Identification

The guts of 30 *T*. *leucotreta* larvae were screened for yeast, which resulted in the isolation of six yeast species. The isolated yeast species were successfully identified via the gene sequence analysis of the ITS region and D1/D2 domain of the LSU (Table 1). The occurrence of the isolated yeast species was not consistent, and the isolated species were not uniformly found in all larvae.

Three yeast species were isolated only from *T*. *leucotreta* larvae collected from Addo, viz. *Meyerozyma guilliermondii*, *Hanseniaspora uvarum* and *Clavispora lusitaniae*. *Kluyveromyces marxianus* was only isolated from a larva collected in Stellenbosch, and the remaining two yeast species, *Pichia kudriavzevii* and *P*. *kluyveri,* were common in larvae collected from each region.

### 3.2. Larval Development Assays

Navel oranges treated with yeast significantly decreased the larval development period and increased the larval pupation rate compared to sterilised fruit with no yeast treatment (Table 2). *Pichia kluyveri* (*p* = 0.0001), *H*. *uvarum* (*p* = 0.0092) and *S*. *cerevisiae* (*p* = 0.0024) performed the best out of all the applied yeast treatments, as not only was development significantly faster than that of the control at a 99% probability level, but pupation success was highest for these three species. Although *P*. *kudriavzevii* (*p* = 0.0586), *K*. *marxianus* (*p* = 0586) and *C*. *lusitaniae* (*p* = 0.002) also significantly decreased the larval development period at the same probability level, the number of pupated larvae was similar to that of the control. Additionally, although not recorded, it was noted that the occurrence of mould was lower on yeast-inoculated Navel oranges compared to the untreated control.

**Figure 2:**
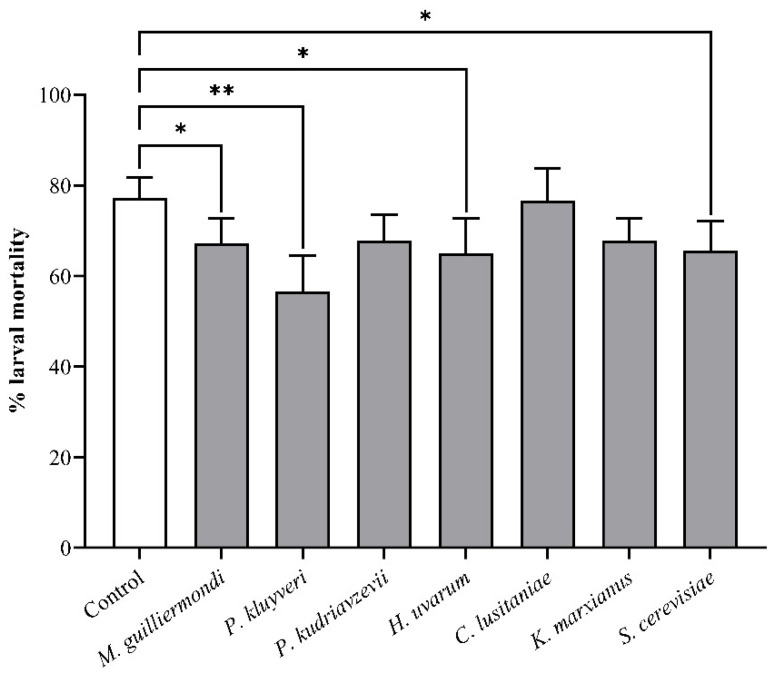
The mortality of *T*. *leucotreta* larvae, determined after 35 d on sterilised Navel oranges (*n* = 36), inoculated with yeast or left untreated. Data are shown as mean ± SE. * and ** indicate significant differences from the control according to a Fisher’s exact test (*p* < 0.05 and *p* < 0.01, respectively).

The mortality rate of *T*. *leucotreta* larvae was significantly lower on Navel oranges treated with *M*. *guilliermondii* (*p* = 0.0452), *P*. *kluyveri* (*p* < 0.0001), *H*. *uvarum* (*p* = 0.0144) and *S*. *cerevisiae* (*p* = 0.0194) (Figure 2). *Clavispora lusitaniae* (*p* > 0.9999), *P*. *kudriavzevii* (*p* = 0.0586) and *K*. *marxianus* (*p* = 0.0586) did not decrease larval mortality. Overall, *P*. *kluyveri* provided the most benefit to *T*. *leucotreta* larvae, as the yeast decreased larval mortality by 20.55% and 74% of larvae pupated before 25 d.

### 3.3. Larval Feeding Assay

**Figure 3:**
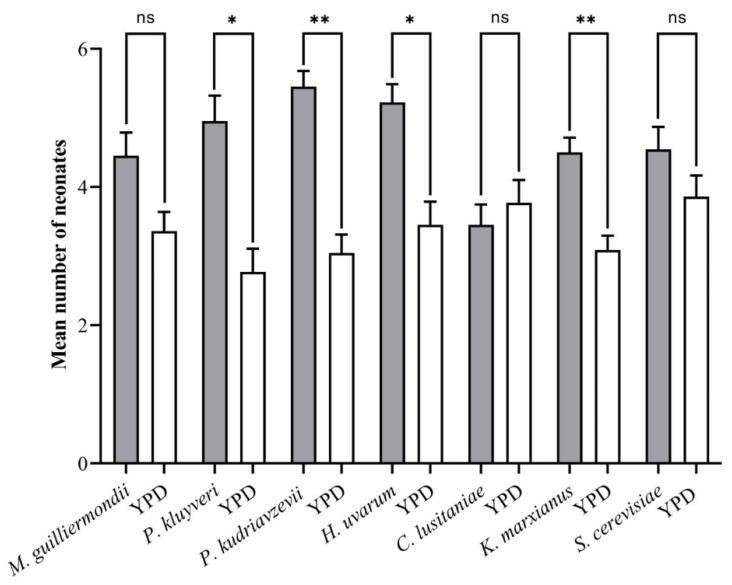
The attraction and feeding of neonate *T*. *leucotreta* in response to yeasts (*n* = 22). * and ** indicate significant differences from the control (YPD) according to a Student’s *t*-test (*p* < 0.05 and *p* < 0.01, respectively); ns indicates that differences were not significant.

The feeding preference of neonate *T*. *leucotreta* was influenced by four yeasts, viz. *P*. *kluyveri* (*p* = 0.0043), *H*. *uvarum* (*p* = 0.0037), *P*. *kudriavzevii* (*p* = 0.0001) and *K*. *marxianus* (*p* = 0.0005) (Figure 3). Two yeast species, *M*. *guilliermondii* (*p* = 0.0606) and *S*. *cerevisiae* (*p* = 0.2579), had no significant effect on larval feeding. The only time larvae preferred the YPD medium was in the case of *C*. *lusitaniae* (*p* = 0.5907).

### 3.4. Oviposition Preference Assays

**Figure 4:**
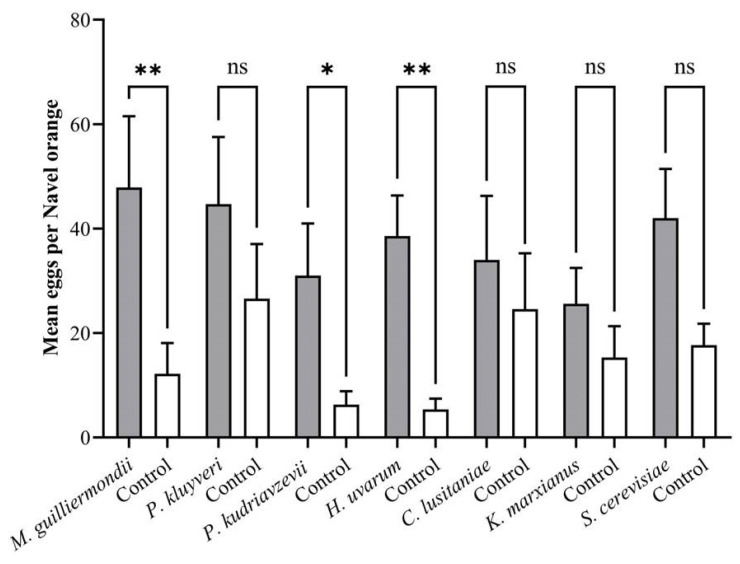
The oviposition preference of adult *T*. *leucotreta* females for yeast during two-choice tests (*n* = 10). * and ** indicate significant differences from the control according to a Student’s *t*-test (*p* < 0.05 and *p* < 0.01, respectively); ns indicates that differences were not significant.

Significantly more eggs were oviposited on Navel oranges inoculated with *M*. *guilliermondii* (*p* = 0.0090), *P*. *kudriavzevii* (*p* = 0.0471) and *H*. *uvarum* (*p* = 0.0013) compared to the untreated control fruit during the two-choice tests (Figure 4). *Pichia kluyveri* (*p* = 0.3768), *C*. *lusitaniae* (*p* = 0.5729), *K*. *marxianus* (*p* = 0.2838) and *S*. *cerevisiae* (*p* = 0.0517) did not influence the oviposition preference of adult *T*. *leucotreta* females.

## 4. Discussion

Most interactions between insects and microorganisms have been studied for their potential use as insect pathogens and not for their role as mutualists [43]. Yeasts play an essential role in the nutrition physiology and host attraction of many insects [19]. The volatile profiles produced by yeasts have also been shown to elicit strong behavioural responses in both lepidopteran larvae and adults [26,41,44]. The potential value of incorporating mutualistic yeast into current strategies to control insect pests has only recently been explored, leading to new pest management strategies [27,28,29]. As this is still a developing area of research, little is known about the influence of naturally occurring yeasts on *T*. *leucotreta*.

*Meyerozyma guilliermondii*, *P*. *kudriavzevii*, *P*. *kluyveri* and *H*. *uvarum* are widely distributed in the environment and often associated with citrus fruits or fermented food [45,46,47,48], so their association with *T*. *leucotreta* is not unexpected. *Hanseniaspora uvarum* is also thought to have a mutualistic relationship with *Drosophila suzukii* (Matsumura) (Diptera: Drosophilidae), a significant pest of soft summer fruits elsewhere in the world [49,50,51,52]. Larval development assays demonstrated that the isolated yeasts have a beneficial effect on *T*. *leucotreta* larvae by reducing their development periods and mortality. There is growing support that the vast majority of insects feed and benefit from yeast in their diet. Yeast multi-cultures have been shown to improve insect development [19]. The composition of an insect’s diet has been shown to affect their adult life traits, including food preference and host locating [53,54]. During larval development, a limited yeast supply can negatively affect adult food preference, copulation, fecundity and longevity [14,53]. In *Drosophila* species, larval diets containing mixed yeast cultures provide more significant development speed and survivability benefits than single yeast cultures [55]. The yeast diet of juvenile insects directly influences their fitness and development and indirectly affects adult life traits [14,53].

Detrimental fungal infections were observed less frequently on Navel oranges treated with yeast than those without, during larval development assays. *Kluyveromyces marxianus* has been reported to be an effective biological control agent against *Penicillium digitatum*, a fungus that causes green mould in citrus fruit, in combination with sodium bicarbonate (NaHCO_3_) [56]. *Hanseniaspora uvarum,* in combination with phosphatidylcholine, has also shown great potential as a biological control agent against *P*. *digitatum* [57]. *Meyerozyma guilliermondii* has also exhibited potential as a biological control agent against fungi responsible for postharvest spoilage of fruit and vegetables [58]. *Thaumatotibia leucotreta*-infested fruit often do not rot, or rotting is delayed, particularly when compared to *Ceratitis capitata* (Wiedemann) (Diptera: Tephritidae) (Mediterranean fruit fly) infestations, indicating that *T*. *leucotreta* larvae may feed on secondary fungal infections, preventing, or delaying, the fruit’s decay. A similar observation has been made in the case of *C*. *pomonella* larval feeding galleries, which rarely become infested with mould [26]. This stands to reason, as the standard artificial diet for laboratory rearing of *T. leucotreta* used to be a *Mucor* or *Rhizopus* fungal species-inoculated maise meal diet [59,60].

Integrated pest management programmes in South Africa successfully use both semiochemicals and pathogens to manage *T*. *leucotreta* populations [33,36,61]. Combining semiochemicals with pathogens in attract-and-kill strategies could significantly increase the efficacy of currently used biological control agents [62,63]. The behaviour of neonate *T*. *leucotreta* was influenced by *H*. *uvarum*, *P*. *kluyveri*, *P*. *kudriavzevii* and *K*. *marxianus*. Volatiles produced by yeasts are thought to stimulate larval feeding [27]. Insect larvae have been shown to prefer specific yeast species over others during the early stages of development [54,64]. In larval feeding assays, it has been demonstrated that *Spodoptera littoralis* (Boisduval) (Lepidoptera, Noctuidae) larvae preferred phyllosphere yeasts over yeasts associated with fruit and frugivorous insects [41]. Increasing larval feeding with yeast increases the probability that a simultaneously applied biopesticide will be ingested by the insect [27,28,29].

*Meyerozyma guilliermondii*, *P*. *kudriavzevii* and *H*. *uvarum* were shown to significantly affect adult *T*. *leucotreta* females’ oviposition preference. Both *H*. *uvarum* and *P*. *kudriavzevii* have now been shown to influence *T*. *leucotreta* neonate and adult female behaviour. They are known to be associated with citrus; thus, their influence on *T*. *leucotreta* is not unexpected. To date, the behavioural influences of these yeasts on lepidopteran moths have not been well documented. However, their influence on other agriculturally important pests has been reported. *Hanseniaspora uvarum* has been shown to affect the ovipositional behaviour of drosophilid flies, acting as both an attractant and repellant [50,65,66]. Using *H*. *uvarum* as a live yeast bait for a selective lure against *D*. *suzukii* has been suggested [67]. The odour profiles of host plants are comprised of numerous volatile organic compounds in a specific blend ratio [68]. Microorganisms can modify this odour profile produced by plants [44]. Moreover, the metabolites produced by gut-associated microbes can affect insects foraging and oviposition behaviour [25]. Volatiles produced by yeasts can elicit a strong response in neonates and adult insects [14,26,41,53]. Host finding and discrimination for *C. pomonella* are mediated by the yeasts’ volatile signatures [26]. In contrast to vision and contact, olfactory cues can be detected at a considerable distance from the source [66,69].

Six yeast species were successfully isolated and identified from *T*. *leucotreta* larvae collected from geographically distinct citrus-producing regions. Larval feeding and oviposition assays demonstrated that the isolated yeast species elicit behavioural responses in *T*. *leucotreta* neonates and adult females. The results generated from this work may help further the development of environmentally safe semiochemicals for population monitoring and control of *T*. *leucotreta* in South Africa. Future work arising from this study would involve conducting detached fruit bioassays and semi-field trials to evaluate the efficacy of a combination with biological control agents currently used against *T*. *leucotreta*.

## Figures and Tables

**Table 1:** BLAST analysis of the ITS region and D1/D2 domain for each isolated yeast species.

Sample	Amplicon	Yeast Species	Accession Number	Identity
Addo “One”	ITS	*Meyerozyma guilliermondii*	MN537824.1	100%
	D1/D2	*Meyerozyma guilliermondii*	EU285513.1	100%
Addo “Two”	ITS	*Hanseniaspora uvarum*	MN371907.1	98.28%
	D1/D2	*Hanseniaspora uvarum*	AY305681.1	99.64%
Addo “Three”	ITS	*Clavispora lusitaniae*	KP765042.1	99.52%
	D1/D2	*Clavispora lusitaniae*	MG871742.1	99.82%
Stellenbosch	ITS	*Kluyveromyces marxianus*	KX376261.1	99.70%
	D1/D2	*Kluyveromyces marxianus*	CP009307.2	100%
Common “One”	ITS	*Pichia kluyveri*	KM982973.1	99.75%
	D1/D2	*Pichia kluyveri*	MN464128.1	99.64%
Common “Two”	ITS	*Pichia kudriavzevii*	LC389027.1	100%
	D1/D2	*Pichia kudriavzevii*	MF461295.1	100%

**Table 2:** The pupation rate of *T*. *leucotreta* larvae before or after 25 d, during 35 d detached fruit bioassays. Sterilised Navel oranges (*n* = 36) were inoculated with yeast or left untreated. * and ** indicate significant differences from the control according to a Fisher’s exact test (*p* < 0.05 and *p* < 0.01, respectively).

Treatments	Total Pupated	Pupated		Percentage before 25 d	*p*-Value
		Before 25 d	After 25 d		
Control	41	15	26	37%	
*M. guilliermondi*	59	35	24	59%	0.0414 *
*P. kluyveri*	78	58	20	74%	0.0001 **
*P. kudriavzevii*	58	35	23	60%	0.0253 *
*H. uvarum*	63	40	23	63%	0.0092 **
*C. lusitaniae*	42	30	12	71%	0.0020 **
*K. marxianus*	58	38	20	66%	0.0075 **
*S. cerevisiae*	62	42	20	68%	0.0024 **

## Data Availability

The data presented in this study are available on request from the corresponding author.

## Supplementary Materials

The following are available online at https://www.mdpi.com/article/10.3390/insects13030243/s1, Figure S1: Feeding response of neonate *T*. *leucotreta* (*n* = 29) to YPD medium containing either (A) red or (B) blue food colourant (*P* > 0.05; t = 0.2133; df = 27 (Students *t*-test)). Images captured by David Taylor, Table S1: Oligonucleotides used to amplify and sequence the internal transcribed spacer (ITS) region and D1/D2 domain of large subunit (LSU) on yeast genomes.
