# Supplementary material for: Mutualism between Gut-Borne Yeasts and Their Host, Thaumatotibia leucotreta, and Potential Usefulness in Pest Management
Source: Insects. 2022 Feb 28;13(3):243. doi: 10.3390/insects13030243 (PMC8954841; doi:10.3390/insects13030243)
Supplement: Supplementary file 1 [file insects-13-00243-s001.zip › insects-1571304-supplementary.pdf]

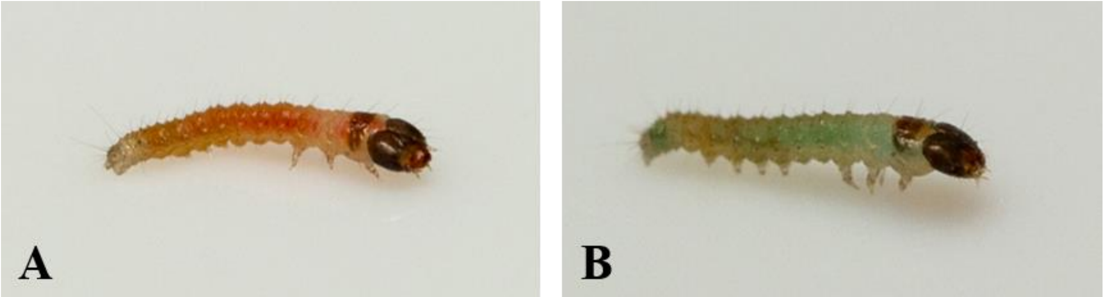

Figure S1: Feeding response of neonate *T. leucotreta* (n = 29) to YPD medium containing either (**A**) red or (**B**) blue food colourant ($P > 0.05$; t = 0.2133; df = 27 (Student's t-test)). Images captured by David Taylor.

Table S1: Oligonucleotides used to amplify and sequence the internal transcribed spacer (ITS) region and D1/D2 domain of large subunit (LSU) on yeast genomes (Pincus et al., 2007).

Target	Name	Sequence (5′ – 3′)	Amplicon size (bp)
ITS region	ITS1	TCC GTC GGT GAA CCT GCG G	400 – 800
	ITS4	TCC TCC GCT TAT TGA TAT GC	
D1/D2 domain	NL 1	GCA TAT CAA TAA GCG GAG GAA AAG	600
	NL 4	GGT CCG TGT TTC AAG ACG G	
